# Supplementary material for: Performance of Serum Angiotensin-Converting Enzyme in Diagnosing Sarcoidosis and Predicting the Active Status of Sarcoidosis: A Meta-Analysis
Source: Biomolecules. 2022 Sep 30;12(10):1400. doi: 10.3390/biom12101400 (PMC9599650; doi:10.3390/biom12101400)
Supplement: Supplementary file 1 [file biomolecules-12-01400-s001.zip › Figure S1.pdf]

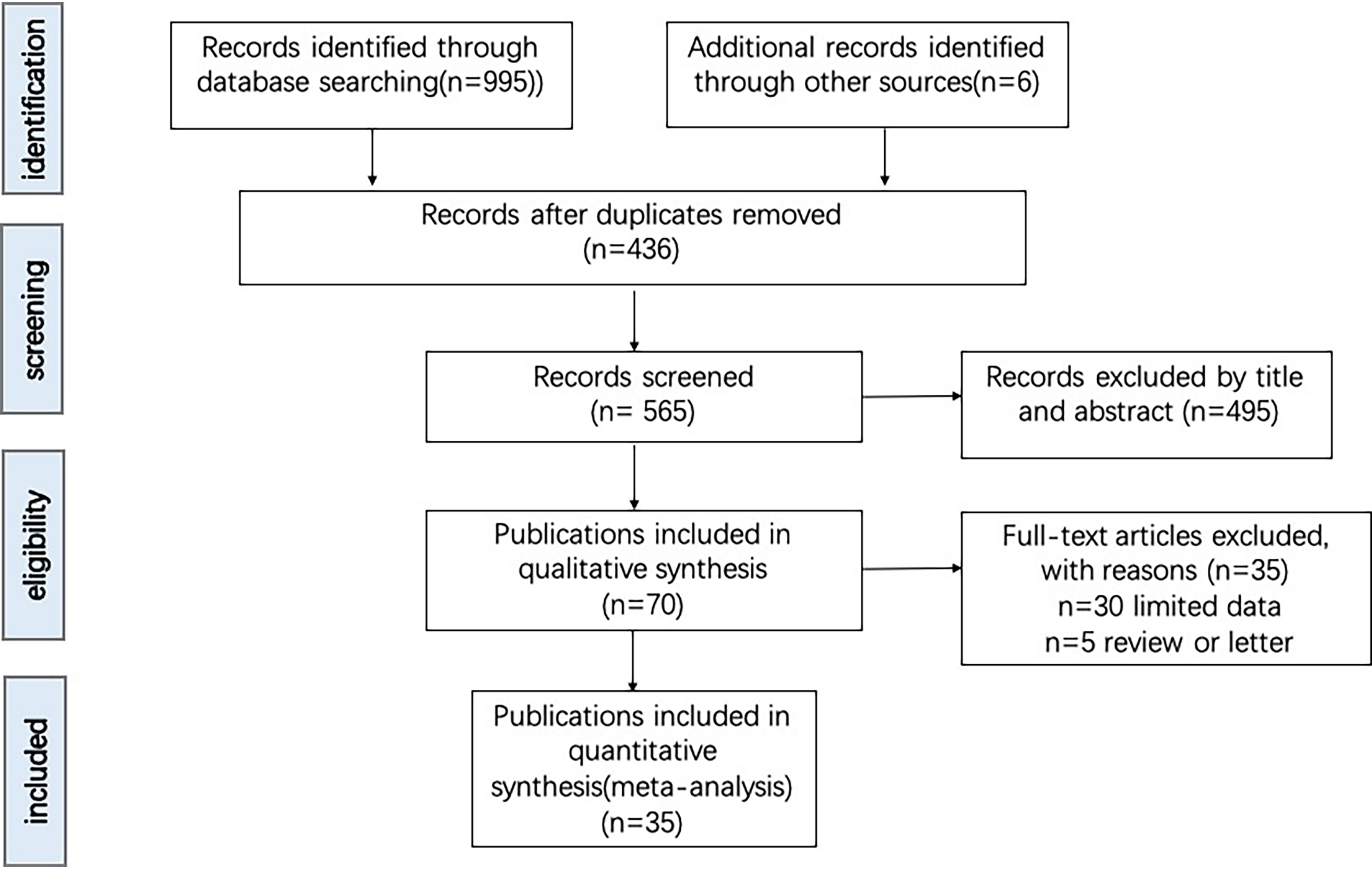
identification
screening
eligibility
included
Records identified through database searching(n=995))
Additional records identified through other sources(n=6)
Records after duplicates removed (n=436)
Records screened (n= 565)
Records excluded by title and abstract (n=495)
Publications included in qualitative synthesis (n=70)
Full-text articles excluded, with reasons (n=35)
n=30 limited data
n=5 review or letter
Publications included in quantitative synthesis(meta-analysis) (n=35)
