# Supplementary material for: Performance of Serum Angiotensin-Converting Enzyme in Diagnosing Sarcoidosis and Predicting the Active Status of Sarcoidosis: A Meta-Analysis
Source: Biomolecules. 2022 Sep 30;12(10):1400. doi: 10.3390/biom12101400 (PMC9599650; doi:10.3390/biom12101400)
Supplement: Supplementary file 1 [file biomolecules-12-01400-s001.zip › Table S1 .pdf]

Table S1 The clinical summary of included studies for ocular sarcoidosis

Author	Year	Country	Criterial	Diseased region	Method	Sarcoidosis					Control					Design	Cut-off	TP	FP	FN	TN
						Case	Age	Gender(M/F)	sACE	Unit	Control	Age	Gender(M/F)	sACE	Unit						
Baarsma et al [36]	1987	Netherlands	Clinical and biochemical findings.	Ocular sarcoidosis	Spectrophotometry	12	NA	NA	NA	U/L	209	NA	NA	NA	U/L	NA	50	10	10	2	199
Power et al [38]	1995	US	Histology	Ocular sarcoidosis	Spectrophotometry	22	35(22-64)	10/12	NA	U/L	70	39(17-58)	29/41	NA	U/L	R	52	16	12	6	58
Kawaguchi et al [41]	2007	Japan	Histology	Ocular sarcoidosis	NA	60	NA	NA	NA	NA	86	NA	NA	NA	NA	R	NA	35	4	25	82
Gundlach et al [17]	2016	Germany	IWOS	Ocular sarcoidosis	Spectrophotometry	41	NA	NA	NA	U/ml	220	NA	NA	NA	U/ml	R	82	9	1	32	219
Hakan et al [44]	2017	Netherlands	IWOS	Ocular sarcoidosis	Spectrophotometry	37	NA	NA	NA	U/L	212	NA	NA	NA	U/L	R	51	20	64	17	148
Ishihara et al [50]	2020	Japan	Clinical findings and/or histology	Ocular sarcoidosis	Spectrophotometry	52	58.8 ± 15.2	13/39	26.4 ± 9.18	U/L	74	49.6 ± 16.5	40/34	13.5 ± 3.83	U/L	R	NA	23	0	29	74
Suzuki et al [53]	2021	Japan	Histology	Ocular sarcoidosis	ELISA	77	54.6 ± 14.8	NA	NA	U/L	79	43.2 ± 17.8	NA	NA	U/L	R	12.7	29	2	48	77
Papasavvas et al [54]	2021	Switzerland	IWOS	Ocular sarcoidosis	Spectrophotometry	37	54.52 ± 23.74	NA	49.17± 29	U/L	30	41 ± 11.3	NA	27.4 ± 15.34	U/L	R	NA	10	1	27	29
