# Supplementary material for: Performance of Serum Angiotensin-Converting Enzyme in Diagnosing Sarcoidosis and Predicting the Active Status of Sarcoidosis: A Meta-Analysis
Source: Biomolecules. 2022 Sep 30;12(10):1400. doi: 10.3390/biom12101400 (PMC9599650; doi:10.3390/biom12101400)
Supplement: Supplementary file 1 [file biomolecules-12-01400-s001.zip › Table S2 .pdf]

Table S2 The diagnosis performance of sACE by ethnicity in sarcoidosis patient

Ethnicity	Sensitivity	Specificity	PLR	NLR	DOR	Area under curve (95% CI)
Caucasian	63%	92%	8.3	0.4	21	0.87(0.84-0.90)
Asian	53%	95%	11.1	0.5	22	0.67 (0.62-0.71)

sACE: serum Angiotensin-Converting Enzyme; PLR: positive likelihood ratio; NLR: negative likelihood ratio; DOR: diagnostic odds ratio; CI: confidence interval
