# Supplementary figures and images for: Performance of Serum Angiotensin-Converting Enzyme in Diagnosing Sarcoidosis and Predicting the Active Status of Sarcoidosis: A Meta-Analysis
Source: Biomolecules. 2022 Sep 30;12(10):1400. doi: 10.3390/biom12101400 (PMC9599650; doi:10.3390/biom12101400)

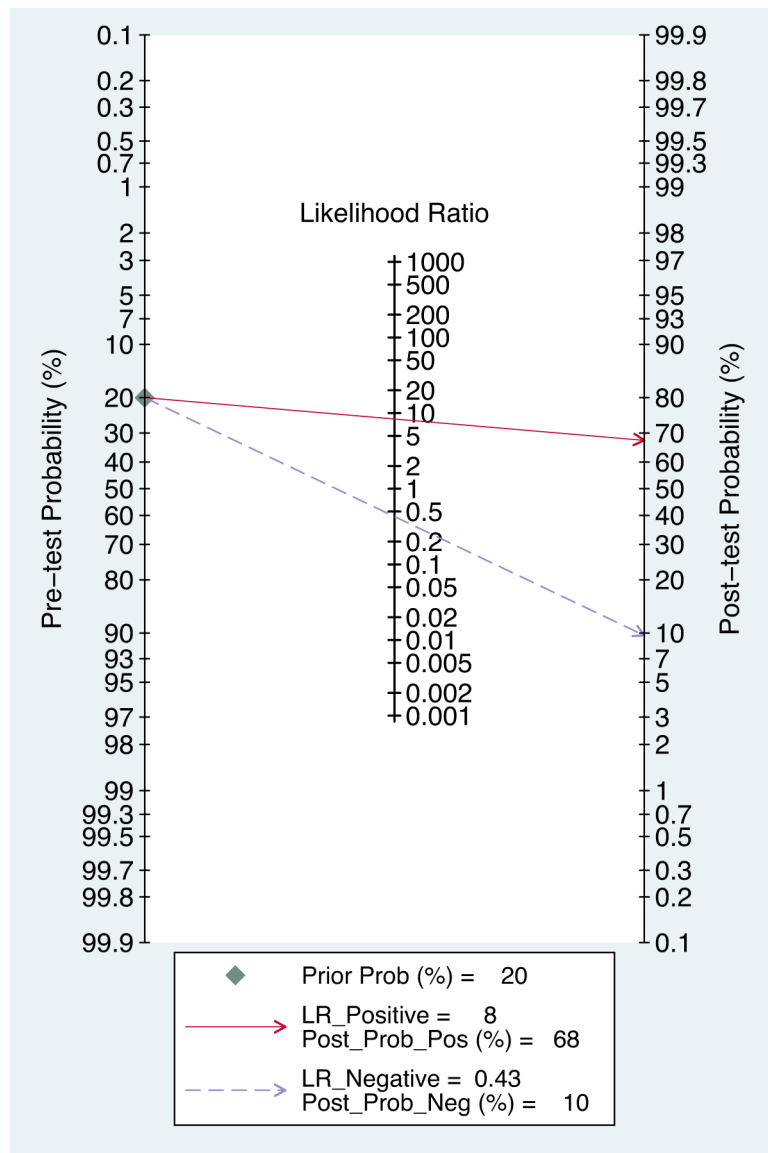

Supplement: Supplementary file 1 [file biomolecules-12-01400-s001.zip › Figure S2.pdf]

## Univariable Meta-regression & Subgroup Analyses

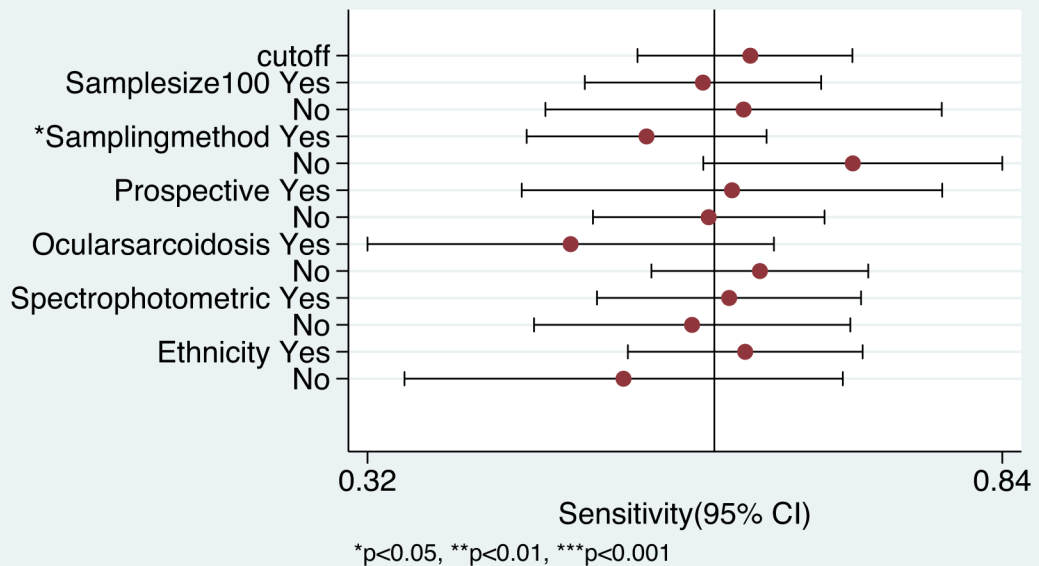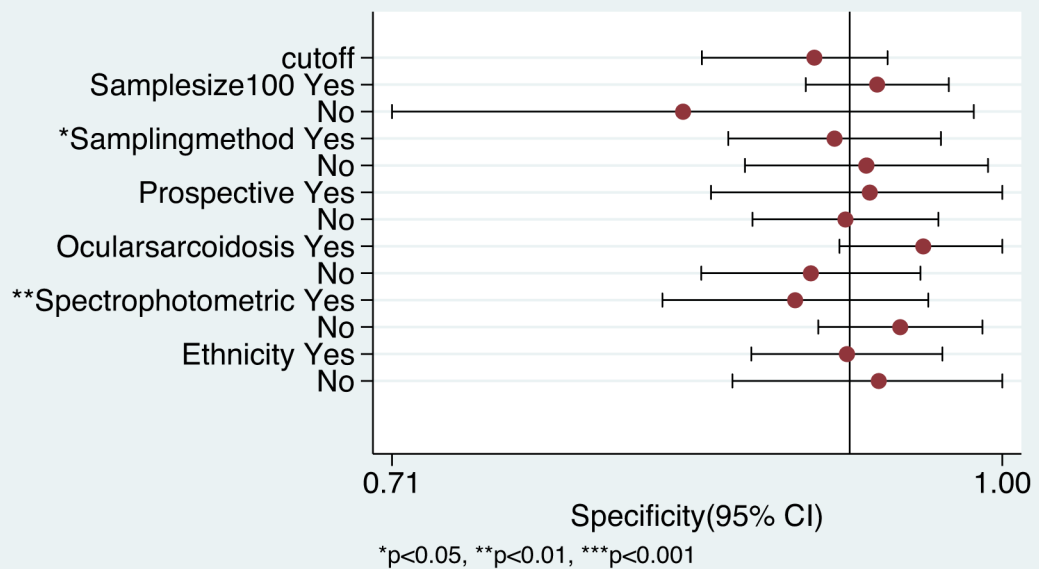

Supplement: Supplementary file 1 [file biomolecules-12-01400-s001.zip › Figure S3.pdf]

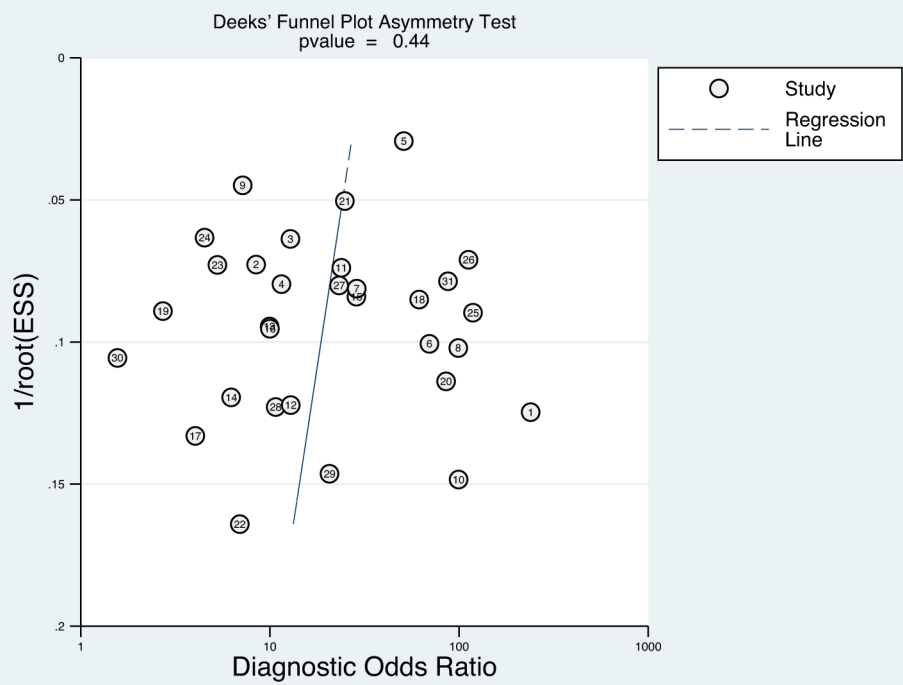

Supplement: Supplementary file 1 [file biomolecules-12-01400-s001.zip › Figure S4.pdf]

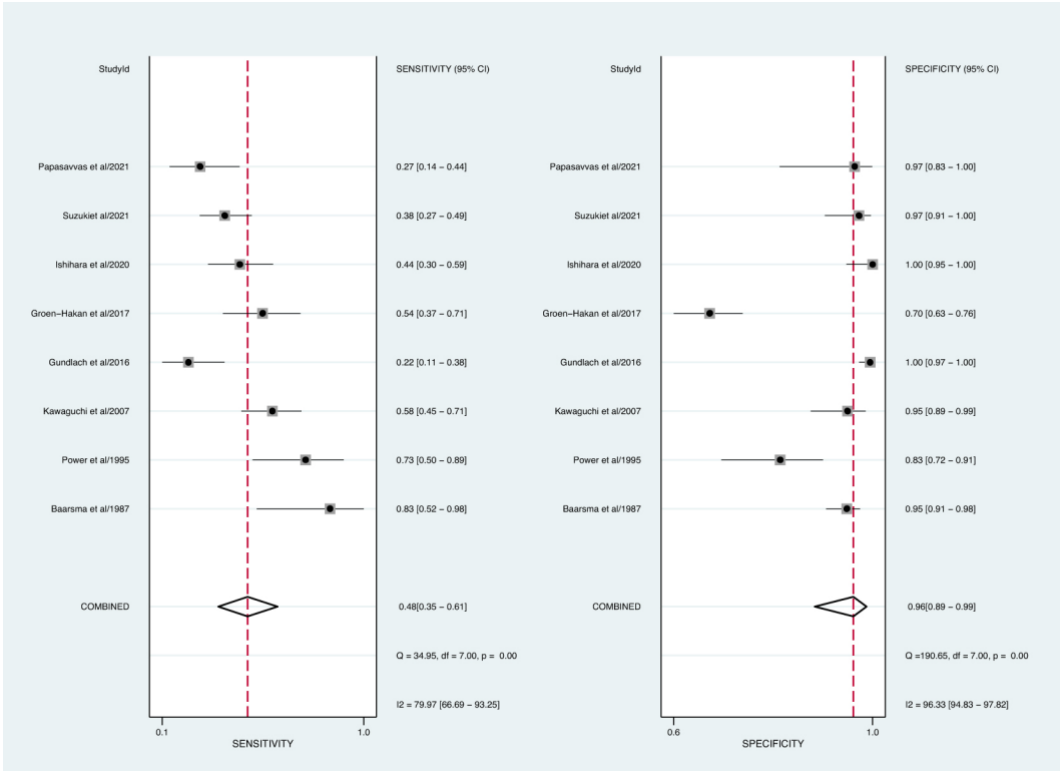

Supplement: Supplementary file 1 [file biomolecules-12-01400-s001.zip › Figure S5.pdf]

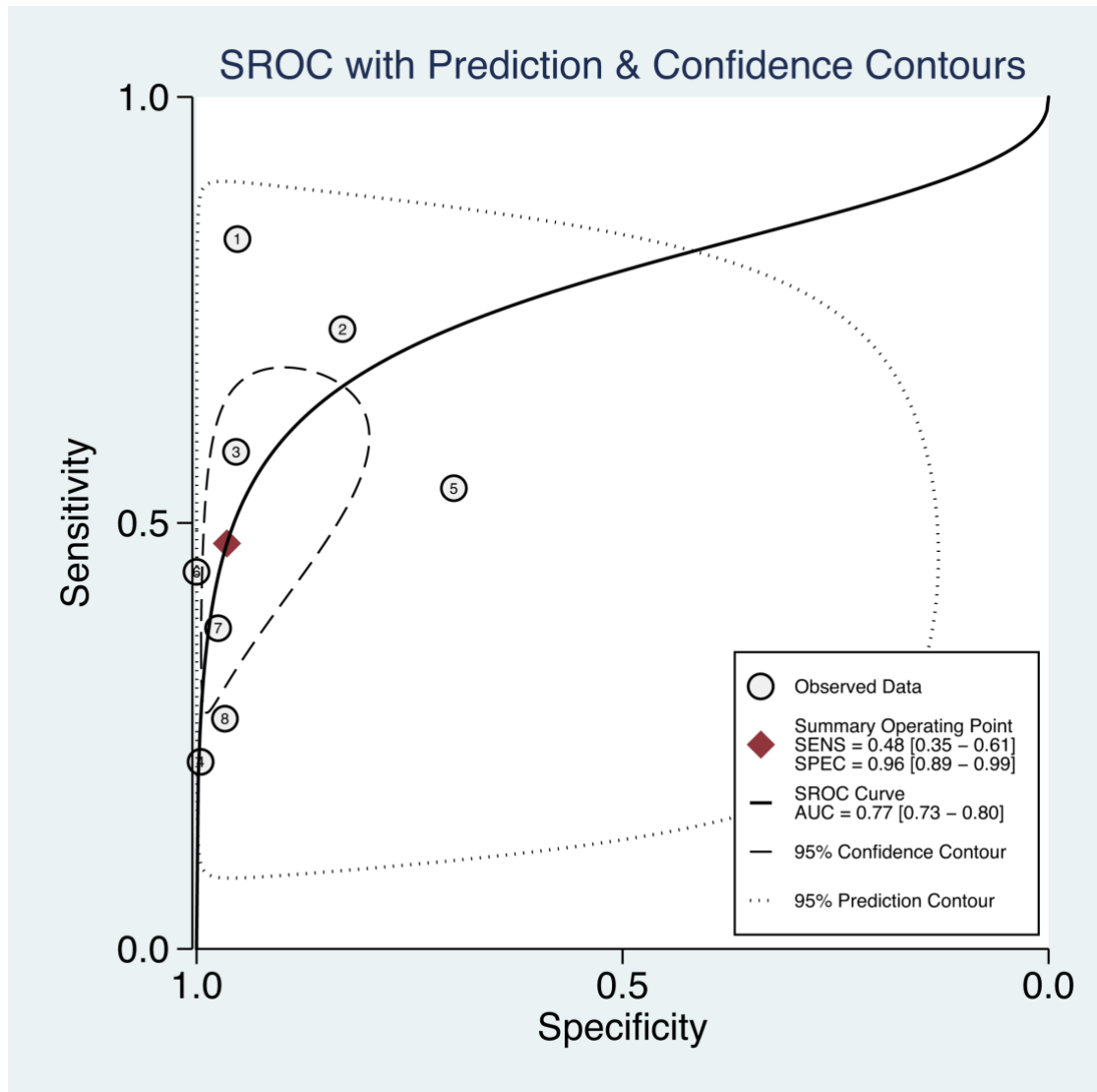

Supplement: Supplementary file 1 [file biomolecules-12-01400-s001.zip › Figure S6.pdf]
